# The RNA-Recognition Motifs of TAR DNA-Binding Protein 43 May Play a Role in the Aberrant Self-Assembly of the Protein

**DOI:** 10.3389/fnmol.2018.00372

**Published:** 2018-10-09

**Authors:** Elsa Zacco, Stephen R. Martin, Richard Thorogate, Annalisa Pastore

**Affiliations:** ^1^UK Dementia Research Institute, King’s College London, London, United Kingdom; ^2^The Maurice Wohl Clinical Neuroscience Institute, Institute of Psychiatry, Psychology and Neuroscience, King’s College London, London, United Kingdom; ^3^The Francis Crick Institute, London, United Kingdom; ^4^London Centre for Nanotechnology, Faculty of Mathematical and Physical Sciences, University College London, London, United Kingdom

**Keywords:** aggregation, ALS, FTLD, RNA-recognition motifs, TDP-43

## Abstract

The TAR DNA-binding protein 43 (TDP-43) is a nucleic acid-binding protein implicated in gene regulation and RNA processing and shuffling. It is a ribonuclear protein that carries out most of its functions by binding specific nucleic acid sequences with its two RNA-recognition motifs, RRM1 and RRM2. TDP-43 has been identified in toxic cytosolic inclusions in neurodegenerative diseases such as amyotrophic lateral sclerosis (ALS) and frontotemporal lobar degeneration with ubiquitin-positive inclusions (FTLD-U). The unstructured C-terminus has prion-like behavior and has been considered the driver of the aberrant self-assembly of TDP-43. In this work, we set out to test the hypothesis that the RNA-binding domains could also play a role in protein aggregation. This knowledge could be of important value for understanding TDP-43 aberrant, disease-leading behavior and, in the future, inform the design of small molecules that could prevent or slow down protein aggregation by exploiting the RNA-binding properties of the protein. We investigated the behavior of the two tandem RRM domains separately and linked together and studied their self-assembly properties and RNA-binding ability with a number of biophysical techniques. The picture that emerges from our study suggests that this region of the protein plays an important and so far unexplored role in the aggregation of this protein.

## Introduction

Amyotrophic lateral sclerosis (ALS) and frontotemporal lobar degeneration (FTLD) are diseases linked clinically, pathologically and mechanistically, and are now recognized as two extreme representatives of a continuum spectrum of the same neurodegenerative disorder (Ferrari et al., 2011). The overlapping molecular features of ALS and FTLD have been associated in post-mortem brains with the presence of cytosolic inclusions of the TAR DNA-binding protein 43 (TDP-43) (Cairns et al., 2007; Sreedharan et al., 2008).

The connection between neurodegeneration and protein misfolding and aggregation has been established over the past 20 years, although the molecular and cellular events leading to aberrant protein self-assembly are as yet unclear (Holmes and Diamond, 2012). Recently, a leading role of nucleic acids in protein aggregation has emerged: aggregation of RNA-binding proteins can be triggered by defective transcription, which would affect RNA processing and, consequently, lead to progressive cell death (Fiesel and Kahle, 2011). A direct effect of RNA binding has also been reported as a possible cause of the aggregation of RNA-binding proteins such as TDP-43. The effect is however, hotly debated: some authors have suggested that formation of cytotoxic aggregates of TDP-43 may be prevented by the interaction with the DNA/RNA binding partner or with its own 3′-untranslated region (3′-UTR) (Pesiridis et al., 2011; Sun et al., 2014); others have reported instead that RNA could force TDP-43 to adopt highly toxic misfolded conformations (Wang et al., 2013). Despite the contradictory evidence, it is indisputable that RNA binding plays a critical role in both TDP-43 non-pathological function and aberrant folding. The toxicity of TDP-43 aggregates may be the result of non-native conformations of the protein leading to a loss of function (Ross and Poirier, 2004): this scenario would result from poor availability of functional TDP-43 in the nucleus, due to the sequestration of the misfolded protein in the cytoso (Xu, 2012). Intrinsically unfolded regions can increase the occurrence of misfolding and can act as prions to push the structural equilibrium toward the unfolded state (March et al., 2016). It has also been reported that the aggregates formed by TDP-43 are not completely amorphous but share properties with amyloid fibrils (Bigio et al., 2013; Mompeán et al., 2016).

TAR DNA-binding protein −43 is a modular protein which contains an N-terminal domain with a nuclear localization signal (NLS) which is thought to play a role in the inter- and intra-molecule interactions, promoting TDP-43 oligomerization and increasing the possibility of the C-terminus driving pathological aggregation (Chang and Huang, 2016; Tsoi et al., 2017). The interaction of the protein with DNA and RNA is mostly achieved by the two RNA-recognition motifs, RRM1 and RRM2, which play a critical role in the TDP-43 functions, including gene regulation and transcription, pre-mRNA editing, microRNA processing and mRNA transport in and out the nucleus (Cohen et al., 2011; Sephton et al., 2012). RRM, also known as ribonucleoprotein (RNP) motif, is a domain of 80–90 amino acids containing two highly conserved consensus sequences (RNP1 and RNP2) of 8 and 6 residues, respectively (Maris et al., 2005). The RRM structure comprises two alpha-helices and four strands assembled in a β-sheet according to a β-α-β-β-α-β topology (Nagai et al., 1990). RNA binding is mostly achieved by the β-sheet and the connected flexible loops which provide the necessary versatility to recognize quite different RNA partners. RRM domains are present, in single or multiple copies, in a vast number of proteins involved in all aspects of RNA processing and transport. TDP-43 association with RNA is also an important self-regulatory process by which the protein controls its own gene expression at the translational level through negative feedback loops (Avendaño-Vázquez et al., 2012). Thousands of RNA sequences have been found to associate with the two RRM domains of TDP-43, mostly localized within intronic regions, 3′-UTRs and non-coding RNA. Most of these sequences share the characteristics of being single stranded and UG-rich, although also non-UG-rich sequences have been identified to interact with TDP-43 to a lesser degree (Sephton et al., 2011; Tollervey et al., 2011; Xiao et al., 2011). It is also important to note that both RRM domains of TDP-43 are not required for RNA binding: it was reported that RRM1 is essential and sufficient for nucleic acid recognition, but not RRM2, which has a weaker affinity for the cognate RNA (Buratti and Baralle, 2001; Kuo et al., 2009). Further adaptability to different binding partners might be given by the flexible loop which joins the two domains (Auweter et al., 2006). Finally, the disordered glycine-rich C-terminus hosts almost all of the mutations identified in patients, several of which enhance protein toxicity (Barmada et al., 2010). The structural flexibility of the C-terminus is not required for nucleic acid-binding activity but plays an essential role in protein–protein interaction and alternative splicing (Ayala et al., 2005; Freibaum et al., 2010).

Here, we were interested in understanding the role of the RRM motifs in TDP-43 aggregation. We studied isolated RRM1 and RRM2 of TDP-43 separately and in tandem and characterized them both for their structural features, RNA-binding ability and aggregation properties using complementary biophysical techniques. We aimed at learning more about the interplay between TDP-43 aggregation and its nucleic acid binding ability by exploiting circular dichroism (CD), thioflavin-T (ThT) binding, atomic force microscopy (AFM), and biolayer interferometry (BLI). We observed a profound difference between the properties of the two domains which strongly influences the behavior of their tandem assembly. We also found that the linker between the domains contributes to an increase in the propensity of the tandem protein toward misfolding and aggregation. Our results constitute an important basis for further studies of TDP-43 aggregation.

## Materials and Methods

### Protein Construct Production and Purification

All protein constructs were encoded in a pET-Sumo expression vector containing the kanamycin antibiotic resistance gene. The plasmids were expressed in Rosetta2(DE3) *Escherichia coli* cell strain as proteins fused with a SUMO solubilization tag carrying a 6×His tag. The cultures were allowed to grow overnight at 37°C in a small volume of Luria-Bertani (LB) medium containing 50 μg/ml kanamycin. Cells were then diluted 1:100 in fresh LB medium with 50 μg/ml kanamycin and allowed to grow at 37°C until an optical density of ca. 0.7 at 600 nm was reached. Temperature was set at 18°C and protein expression was induced by 0.5 mM IPTG. Cells were collected after overnight growth by centrifugation at 4000 rcf for 20 min at 4°C, then resuspended in lysis buffer (10 mM potassium phosphate buffer pH 7.2, 150 mM KCl, 5mM imidazole, 5% v/v glycerol, 1mg/ml lysozyme, complete^TM^ EDTA-free Protease Inhibitor tablet by Roche, and 1 μg/ml DNase I and 1 μg/ml RNaseA). Cells were broken by probe-sonication and soluble proteins were recovered by centrifugation at 70000 rcf for 45 min at 4°C. The total soluble protein mixture was loaded onto a column packed with nickel-coated resin and the 6xHis-SUMO-construct was eluted with high-salt phosphate buffer (10 mM potassium phosphate buffer pH 7.2, 150 mM KCl) with the addition of 250 mM imidazole. The eluate was dialyzed overnight at 4°C against low-salt phosphate buffer (10 mM potassium phosphate pH 7.2, 15 mM KCl) in the presence of the Tobacco Etch Virus (TEV) protease at a 1:20 protein construct:TEV molar ratio, to remove the 6×His-SUMO tag. A second nickel-affinity chromatography followed. The flow-through was collected and loaded onto a HiTrap Heparin column to remove unwanted nucleic acids. The protein construct was eluted with potassium phosphate buffer with 1.5 M KCl and submitted to size-exclusion chromatography with a HiLoad 16/60 Superdex 75 prep grade in low-salt phosphate buffer. Protein identity and purity were checked by PAGE and mass spectrometry.

### Circular Dichroism Measurements

Circular dichroism spectra were recorded on a JASCO-1100 spectropolarimeter. The spectra were acquired in 0.1 mm path length quartz cuvettes under a constant N_2_ flush at 4.0 L/min. All CD datasets were an average of ten scans. For the determination of the global secondary structure, the far-UV (190–260 nm) spectrum was recorded at 20°C in low-salt phosphate buffer. Samples were gradually heated to 90°C (3°C/min) for the determination of the melting temperature (Tm). The ellipticity variation as expressed by the intensity of the band at 222 nm was followed as a function of temperature. Variations in the secondary structure were also followed by fixing the temperature at 37°C and acquiring spectra at time 0, after 24 and 48 h. The data were plotted and the Tm was calculated according to literature (Greenfield, 2006). Control CD spectra were acquired after the temperature was brought back to the original 20°C (3°C/min). The spectra were corrected for the buffer signal and expressed as mean residue molar ellipticity θ (deg∗cm^2^∗dmol^−1^).

### ThT Binding Assays

Protein aliquots were rapidly thawed, spun down, and filtered with a 0.2 μm syringe filter before each assay to ensure removal of any contingent pre-formed oligomers. The concentration was then determined to ensure homogeneity of results and all assays were performed by diluting the protein sample to 15 μM in high-salt phosphate buffer and by adding 20 μM ThT. The excitation wavelength was set at 440 nm. Emission was set from 460 to 600 nm for the spectrofluorometer and at 485 nm for the multi-well plate reader.

Information about the nature of the aggregates formed upon heat treatment was obtained by dividing the protein constructs into two aliquots. ThT was added to the first aliquot and the emission fluorescence intensity was determined. The second aliquot underwent heat treatment (3°C/min from 20 to 90°C) then allowed to cool down for 20 min, and only at this point ThT was added and the emission fluorescence intensity was again determined. This protocol allows one to maintain the structural integrity of the ThT molecules, which would have otherwise degraded at higher temperatures. This experiment was performed in a 1-cm quartz cuvette with the Jasco spectrofluorometer FP-8200.

Aggregation kinetics for all constructs were also determined by the ThT-binding assay. Formation of amyloid-like structures was followed at 37°C by recording the fluorescence intensity of ThT as a function of time. The reported data derive from at least three repetitions and are presented as percentage values, where 100% is the highest fluorescence intensity value registered during the assay. Aggregation kinetics were followed using a BMG FLUOstar Omega microplate reader in Greiner Bio-One CELLSTAR plates.

### Biolayer Interferometry Measurements

Experiments were performed in high-salt phosphate buffer with 0.05% Tween on an Octet RED System (Pall ForteBio Corp., Menlo Park, CA, United States) operating at 25°C. RNA molecules (1 μl/ml) were loaded onto streptavidin coated biosensors (FortèBio Dip and Read^TM^) for at least 100 s to saturate the sensors. Binding to the three protein constructs in a concentration range 1 nM – 15 μM was measured for 10–40 min or until plateau was reached. Dissociation was followed for at least 100 s. Dissociation constant (*K*_d_) values were estimated by fitting the response intensity (nm) as a function of protein concentration (μM) with the Octet Data Analysis Software. The RNA sequence used to determine binding affinity (AUG12) was selected according to published data (Lukavsky et al., 2013). The chosen negative control (NegAUG12) is the reverse and complementary of the AUG12 sequence.

### Atomic Force Microscopy

Samples for AFM were recovered directly from the aggregation kinetics assay plate, in which 15 μM protein constructs were incubated at 37°C in high-salt phosphate buffer for 3 days. The samples were 20-fold diluted in water to obtain images from isolated aggregates and 2 μl were loaded onto freshly cleaved mica and left at room temperature for 5 min to allow excess liquid to evaporate.

Height and peak force error images were acquired on a Bruker Dimension Icon microscope with a Nanoscope V controller (Bruker UK Ltd., Santa Barbara, CA, United States) operating in peak force tapping mode using ScanAsyst Air cantilevers (115 μm nominal length, 25 μm nominal width, nominal spring constants of 0.4 N/m). The ScanAsyst probes have 2 nm nominal tip radius of curvature. Image data were acquired at Peak Force frequency of 2 kHz and a line rate of 0.75–1 Hz at a resolution of 256 pixels per line.

## Results

### The Behavior of RRM1-2 Reflects a Tug-of-War Between RRM1 Aggregation Propensity and RRM2 Stability

**FIGURE 1:**
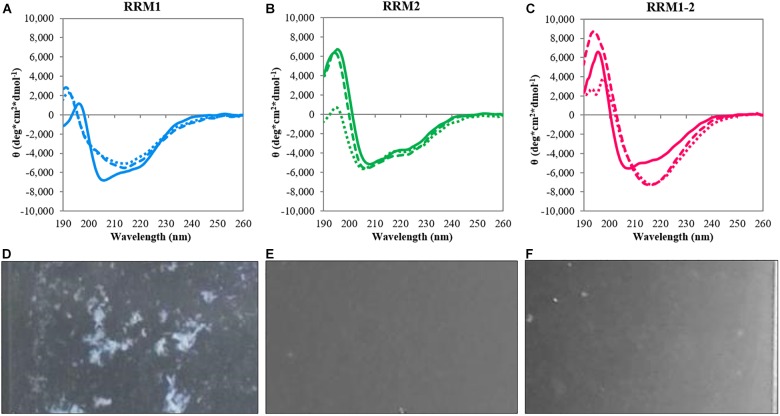
Circular dichroism analysis of the TDP-43 constructs. Top panels: spectra at 20°C (continuous lines), after heat treatment at 90°C (dotted lines) and after reporting the samples at 20°C (dashed lines) for RRM1 **(A)**, RRM2 **(B)**, and RRM1-2 **(C)**. Bottom panels: Camera photos of the CD quartz cuvettes after heat treatment of RRM1 **(D)**, RRM2 **(E)**, and RRM1-2 **(F)** to document the precipitation observed upon heating the TDP-43 samples. RRM1 displays a significant amount of precipitation visible by naked eye. RRM2 appears as a clear solution. RRM1-2 shows a certain degree of cloudiness but no large precipitates are visible.

Circular dichroism spectroscopy was employed to investigate secondary structure, thermal stability, and conformational changes of the TDP-43 portions RRM1 and RRM2 independently and expressed in tandem (RRM1-2) (**Table 1**). All constructs displayed similar CD spectra at 20°C typical of relatively well folded proteins (**Figures 1A–C**). In all cases, we observed two minima at 208 and 222 nm that are indicative of the presence of α-helical structures. Some minor differences were observed in the CD intensity values of the maximum at 195 nm: despite the structural similarity, the spectrum of RRM1 had a significantly less intense low wavelength maximum compared to RRM2 and RRM1-2. This could indicate the contribution of the disordered 15-residues linker at RRM1 C-terminus.

**Table 1:** Summary of the TDP-43 constructs involved in this study.

Name	Amino acid	Description
RRM1	K102-R191	RNA-recognition motif 1 and 15-amino acid linker
RRM2	R191-Q269	RNA-recognition motif 2
RRM1-2	K102-Q269	The two RNA-recognition motifs with their linker

*The table reports names attributed to the protein constructs, sequences corresponding to the amino acid position in the full-length TDP-43 and description. Enumeration refers to the full-length protein.*

To mimic stress conditions, we collected spectra after gradually increasing the temperature up to 90°C. The three constructs had a quite different behavior. The spectra of RRM1 and RRM1-2 had clear transitions toward shapes typical of β-sheet proteins with a single minimum at approximately 218 nm (**Figures 1A–C**). RRM2 remained instead mostly invariant also at the maximal temperature indicating a strong resistance to thermal denaturation (**Figure 1B**). We checked for reversibility by gradually lowering the temperature back to the original 20°C. The spectra of RRM2 before and after heating were the same, indicating full reversibility of the unfolding process. This observation is consistent with a previous report which indicated that RRM2 is more structurally stable (Mackness et al., 2014). Conversely, the spectra of RRM1 and RRM1-2 remained the same as at high temperature with a single minimum around 218 nm. The spectrum of RRM1-2 maintained a maximum at 195 nm which slightly increased in intensity. This effect could reflect the formation of oligomers which might cause scatter of the signal below 200°nm (Greenfield, 1999). Visual inspection of the cuvettes after heating showed visible precipitation of RRM1 but not of the other two samples (**Figures 1D–F**).

**FIGURE 2:**
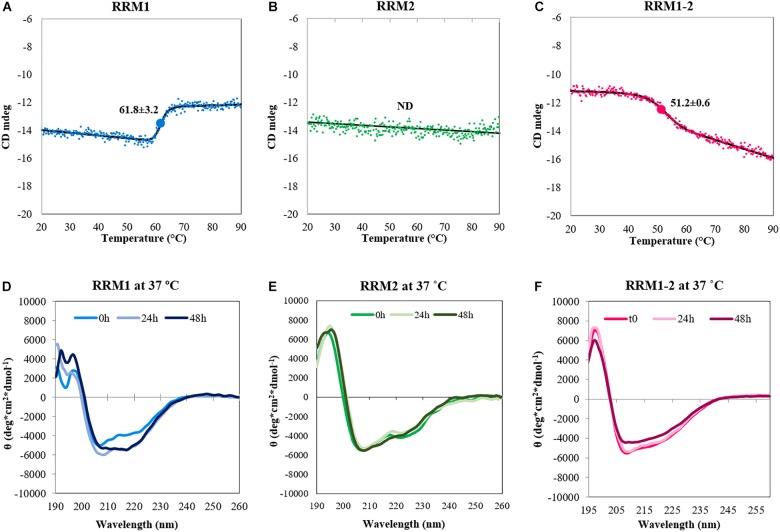
Conformational changes of the TDP-43 constructs upon heat treatment. Top panel: Denaturation curves determined by CD spectroscopy by recording the intensity values of the band at 222 nm as a function of temperature for RRM1 **(A)**, RRM2 **(B)**, and RRM1-2 **(C)**. The spectrum associated to the denaturation of RRM1 cannot be considered reliable, due to the large amount of precipitation observed. The value of the CD signal at 222 nm does not change for RRM2 indicating (together with the data in **Figure 1**) no thermal unfolding. The apparent T_m_ of RRM1-2 is 51.2 ± 0.6°C. Bottom panel: Kinetics of secondary structure variations at 37°C for RRM1 **(D)**, RRM2 **(E)**, and RRM1-2 **(F)**. The data were acquired in low-salt potassium phosphate buffer.

In a different experiment, we performed temperature scans by gradually heating up the samples to 90°C and following the effect on the intensity of the bands at 222 nm (**Figures 2A–C**). Also in this case RRM2 was stable and did not show the typical folded-to-unfolded biphasic transition, resulting in a flat line. The other two constructs showed instead sigmoidal curves with transition midpoints at 61.8 ± 3.2 and 51.2 ± 0.6°C for RRM1 and RRM1-2, respectively. These values should of course be considered only apparent melting points since they represent an irreversible process mediated by aggregation.

Finally, we fixed the temperature at 37°C and followed the behavior of the samples at different times up to 48 h to follow the kinetics of secondary structure variations at near-to-physiological conditions (**Figures 2D–F**). The CD spectrum acquired at time 0 was similar to that recorded at 20°C, but we observed a clear change in secondary structure after 48 h for RRM1. The spectrum acquired a unique minimum at 218 nm that is a feature characteristic of a β-sheet rich secondary structure. RRM1-2 had a similar transition but the changes were less obvious within the 48 h of the experiment. RRM2 maintained its native conformation throughout the duration of the assay.

Taken together, these results suggested that RRM1 has an intrinsically higher tendency to adopt a β-rich conformation than RRM2. The effect seemed to be mitigated in RRM1-2 by RRM2 which has characteristics intermediate between the two isolated RNA-binding motifs. Our evidence also suggests for the first time a potential role of the two RRM motifs in the aberrant self-assembly of TDP-43, at variance with previous reports that have stressed almost exclusively the effect of the disordered C-terminus.

### RRM1 and RRM1-2 Display Amyloid-Like Properties With Different Aggregation Kinetics

**FIGURE 3:**
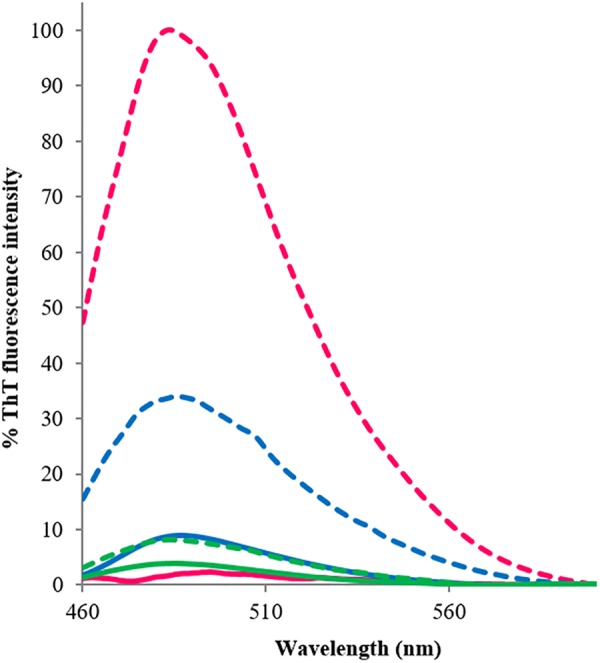
Thioflavin-T emission fluorescence spectra associated with the three TDP-43 constructs, recorded at room temperature at between 460 and 600 nm, before (continuous lines) and after (dashed lines) heat treatment. The excitation wavelength was set at 440 nm. Blue: RRM1; green: RRM2; and fuchsia: RRM1-2. The reported fluorescence values are an average of three experiments, each conducted with three accumulations. The results are expressed as percentage, where 100% is the highest ThT-associated fluorescence value obtained within the assays, corresponding to RRM1-2 emission value at 485 nm after heating.

To establish whether the β-rich structures formed upon heat treatment are amyloid in nature, we exploited the ability of the fluorescent dye ThT to bind preferentially to amyloid-like structures proportionally to the amount of aggregates present in the sample (**Figure 3**). Before heat treatment, the fluorescence signal of RRM1 was negligible. After heating, the signal increased by ca. 4.5 folds. The modest increase in fluorescence of RRM1 could likely be explained by the high degree of precipitation typical of this construct. The portion of the construct remaining in solution nevertheless had amyloid-like properties, while most of the sample aggregated as large floccules and precipitated. By measuring the variation of the concentration in solution before and after heating, we estimated that up to 70% of RRM1 would be lost due to precipitation after heat treatment (data not shown). Under the same protocol, RRM2 showed no significant fluorescence variation, going from a basal 5% intensity to 10%, consistent with the low tendency to self-assemble also when under substantial stress as observed by CD. Finally, the fluorescence intensity of RRM1-2 displayed a 75-fold increment upon heating and no significant visible precipitation suggesting that, although the stabilizing effect of RRM2 when associated with RRM1 strongly contributes to maintaining RRM1-2 in a soluble state, the effect is insufficient to prevent formation of amyloid-like aggregates.

**FIGURE 4:**
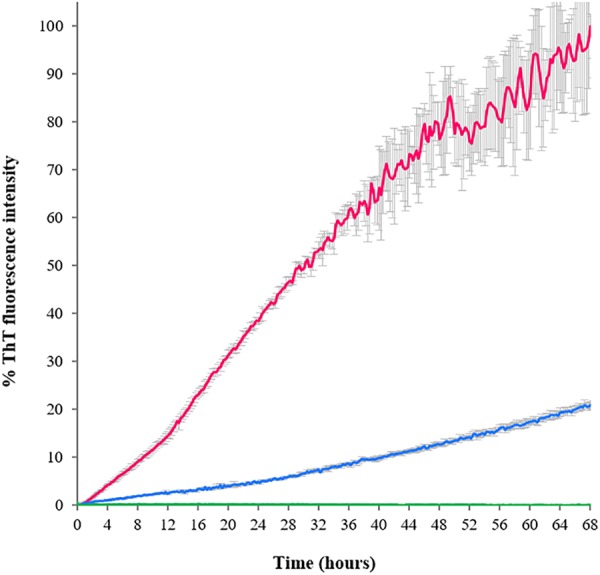
Aggregation kinetics of the TDP-43 constructs determined according to the ThT emission fluorescence at 485 nm as a function of time. Blue: RRM1; green: RRM2; and fuchsia: RRM1-2. The fluorescence values are reported as percentage, where 100% is the highest ThT-associated fluorescence value obtained for the assay, corresponding to RRM1-2 at 68 h.

The ThT-binding assay was also used to establish the aggregation kinetics of the three TDP-43 constructs when left for several hours at 37°C (time scan under nearly physiologic conditions, **Figure 4**). The aggregation curve of RRM1-2 was linear with a near-to-constant pendency. The fluorescence intensity signal gradually and continuously increased for the duration of the assay (68 h). No real plateau was reached but the signal became noisier and more scattered, presumably because of the formation of larger aggregates and eventually their deposition. Determination of protein concentration at the end of the assay indicated that up to 35% of RRM1-2 precipitated. After almost 3 days at 37°C, RRM1 displayed a ThT-associated fluorescence equal to 20% of the one registered for RRM1-2 and a substantial precipitation (up to 95% as judged from concentration quantification). Heavy precipitation, rather than limited aggregation, was probably the real reason for the poor fluorescence signal. RRM2, instead, could be fully recovered in solution and no increment of ThT-associated fluorescence was detected, confirming the ability of this construct to maintain its native, soluble conformation also within the conditions of this assay.

These data are in full agreement with the CD results, confirming a marked different behavior of the two RRM domains.

### RRM1 and RRM1-2 Form Aggregates of Similar Morphology but Different Thickness

**FIGURE 5:**
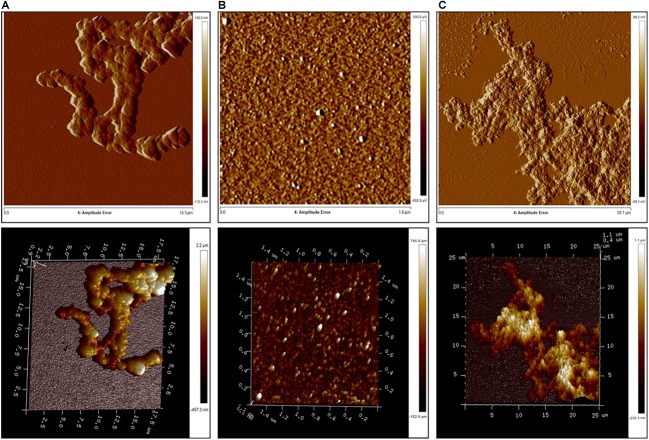
Atomic force microscopy analysis of aggregate morphology. **(A)** RRM1; **(B)** RRM2; and **(C)** RRM1-2.

We then investigated the supramolecular structures formed by RRM1, RRM2, and RRM1-2 by AFM (**Figure 5**). We allowed the protein constructs to fully aggregate and analyzed the resulting formations after 72 h. The aggregates formed by RRM1 appeared compact and uniformly thick, with an average thickness of 365 nm. As expected, RRM2 did not form large aggregates but only what appeared to be either small oligomers or simply crystals formed by the phosphate buffer. The average thickness of these structures was 5.38 nm. RRM1-2 formed different-looking aggregates as compared to RRM1. They appeared softer, almost cotton-like and highly variable in density along their structure, with an average thickness of 173 nm.

Our morphological examination agrees with the CD analysis and ThT-binding assay, since, even if aggregates for RRM1-2 are indeed formed, their composition was not as dense as the ones created by RRM1. These results confirmed that RRM1-2 exhibits an intermediate behavior between RRM1 and RRM2.

### RRM1 Is Necessary but Not Sufficient for Efficient RNA Binding

**FIGURE 6:**
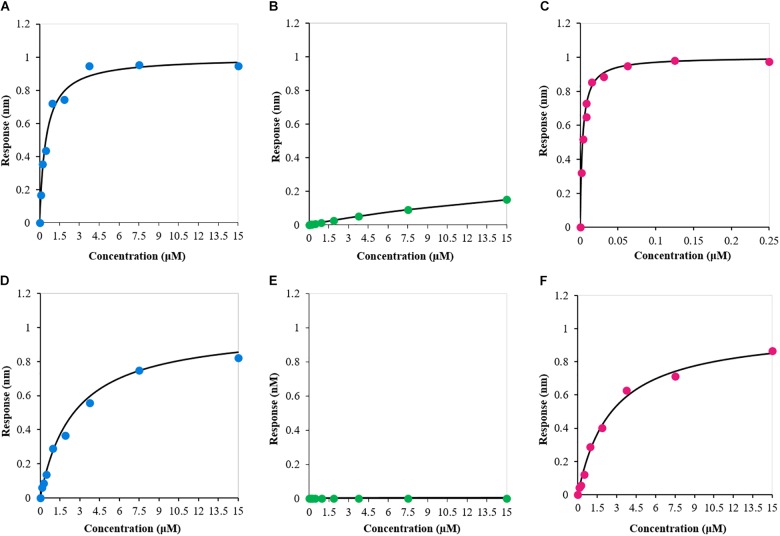
Biolayer interferometry-derived steady-state analysis of the binding response (nm) as a function of protein construct concentration (μM). **(A)** RRM1–AUG12; **(B)** RRM2–AUG12; **(C)** RRM1-2–AUG12; **(D)** RRM1–NegAUG12; **(E)** RRM2–NegAUG12; and **(F)** RRM1-2–NegAUG12.

Finally, we used BLI to investigate the independent contribution of the two RNA-recognition motifs to bind specific RNA molecules and compare the results with those obtained for RRM1-2. To this end, the 12-nucleotide single-stranded RNA sequence AUG12 (5′-GUGUGAAUGAAU-3′), which is known to strongly bind TDP-43 tandem RRM1-2 domains, was selected. We individually analyzed the interaction of the three protein constructs with AUG12 to define the binding affinities of the complexes (**Figures 6A–C**). The dissociation constant (*K*_d_) values determined for the binding pairs RRM1-AUG12 and RRM2-AUG12 were 0.48 ± 0.09 μM and >30 μM, respectively, (**Table 2**, entries 1 and 2). The interaction of RRM1-2 with AUG12 provided a *K*_d_ of 3.9 ± 1.1 nM (**Table 2**, entry 3). These data confirmed the leading role of RRM1 in nucleic acid binding (Mackness et al., 2014), with RRM2 having a *K*_d_ at least 60 times lower than RRM1. The *K*_d_-values also underlined how RRM1 by itself may be necessary but not sufficient to justify the binding affinity of RRM1-2 with UG-rich RNA molecules, which is in the low nanomolar range. Although RRM2 by itself seemed unable to allow appreciable RNA-binding, its presence seems essential for cooperative binding.

**Table 2:** Dissociation constants (*K*_d_) values defined by means of BLI for each binding pair.

Entry	Binding pair	*K*_d_ (μM)
1	RRM1 – AUG12	0.48 ± 0.09
2	RRM2 – AUG12	>30
3	RRM1-2 – AUG12	0.0039 ± 0.0009
4	RRM1 – NegAUG12	2.55 ± 0.48
5	RRM2 – NegAUG12	N.D
6	RRM1-2 – NegAUG12	2.6 ± 0.6

*The values are all expressed in μM for conformity.*

To confirm that the obtained binding constants are specific for UG-rich RNA molecules, we also determined the *K*_d_-value of the three protein constructs with the reverse and complementary sequence of AUG12, 5′-AUUCAUUCACAC-3′, hereafter called NegAUG12 (**Figures 6D–F** and **Table 2**, entries 4–6). RRM1-NegAUG12 displayed a *K*_d_ 5-fold higher than that recorded for the UG-rich RNA, while no *K*_d_ could be determined for RRM2-NegAUG12 under the tested conditions. RRM1-2 still recognized the RNA to some extent, but the *K*_d_-value obtained was 650-fold higher than for the non-UG-rich RNA as compared to AUG12.

These results mirrored what we had observed with AUG12, and emphasized that, despite RRM1-2 retaining the ability to bind non-UG-rich RNAs, it is the selectivity of the recognition for specific sequences that makes the protein capable of carrying out so many different functions.

## Discussion

In this work, we focused on better understanding the role that the two TDP-43 RNA-recognition motifs play in protein aggregation. To our knowledge, ours is the first study focusing on the potential role of the TDP-43 RRM motifs in aggregation. While the isolation of a motif from the full-length protein and other possible interacting partners may not necessarily reflect the complexity of the full-length system, it is clear that RRM1 and RRM1-2 are both able to misfold under stress conditions. We may thus suggest that the two RRM domains of TDP-43 could play an active role in protein aggregation that has so far been under looked.

Approximately 2% of human gene products contain at least one RRM domain which constitutes an important RNA-recognition motif (Malhotra and Sowdhamini, 2014). It is therefore not surprising that misfunction and misfolding of RRM-containing proteins are associated with several neurodegenerative disorders. Among them, the neurological diseases associated with aberrant aggregation of TDP-43 define a novel group of proteinopathies (Arai et al., 2006; Cook et al., 2008). Nevertheless, little attention has been paid up to now to the RRM domains of TDP-43, most of the attention being focused on the C-terminus of the protein which contains most of the pathology-associated mutations.

We examined the behavior of these motifs separately (RRM1 and RRM2) and linked together (RRM1-2) for their structural characteristics, stability under thermal stress, RNA-binding properties and morphology of the aggregates. We found profound differences in their properties despite the high similarity in sequence and structure. First of all, the two domains have different structural stabilities as measured both by measuring kinetics at 37°C and the protein response to heat shock. This is not surprising since it is well known that the CD spectra associated with different RRM motifs can vary among different proteins (Iko et al., 2004) and within proteins containing more than one motif (Lu and Hall, 1995; Kurihara et al., 1997). Kuo et al. (2009) have already hinted at different structural characteristics between RRM1 and RRM2, showing that the latter is more stable and is more resistant to thermal denaturation (Kuo et al., 2009). Their CD analysis on RRM1 shows a heat-induced loss of signal intensity and an increment in β-sheet content. The authors also reported that the construct formed by the two domains together (note that this construct differs only by the addition of one N-terminal amino acid from ours) behaved like RRM1. Furthering this study, our results demonstrated a stabilizing effect of RRM2 on RRM1-2: in our hands, RRM1-2 displayed structural characteristics intermediate between RRM1 and RRM2, forming β-rich aggregates that could be maintained in solution over the time of the experiments, contrary to the high levels of precipitation observed for RRM1 alone at high temperatures. Mackness et al. (2014) also approached RRM1, RRM2, and RRM1-2 by means of CD spectroscopy (Mackness et al., 2014). The authors also reported a stabilizing role of RRM2 but their CD spectrum of RRM1 at 20°C substantially differs from that reported by us and Kuo et al. (2009) with a higher CD intensity as compared to RRM2 and RRM1-2, and a 208/222 nm ratio >1, which indicated a more abundant α-helical content. The discrepancy between these results could easily be explained as the consequence of the absence in Mackness’ construct of the 15-amino acid linker at the C-terminus of RRM1. Linkers between RRM domains are known to have high conformational flexibility that could allow different orientation of the two RNA-binding domains toward their interaction partners (Teplova et al., 2010). Being flexible and partially hydrophobic, the RRM1–RRM2 linker of TDP-43 could thus be instrumental in leading to the aggregation of our RRM1 and RRM1-2 (Lukavsky et al., 2013).

We then set out to study the morphology of the aggregates from the two RRM domains of TDP-43. The nature of the aggregates formed by TDP-43 is at the center of a complex debate in the scientific community. Early studies reported that TDP-43 aggregates extracted from human brains did not show amyloid-like features when observed with an optical microscope and are unable to bind the amyloid-specific dye thioflavin S (ThS) (Cairns et al., 2007; Neumann et al., 2007). In contrast, Thorpe et al. (2008), who approached the issue by exploiting immunoelectron microscopy techniques, asserted that TDP-43 inclusions derived from FTLD patient tissues displayed both granular and filamentous components, firmly suggesting the formation of amyloid-like fibrils in affected neurons (Thorpe et al., 2008). This evidence is supported by studies conducted by Bigio et al. (2013) and Robinson et al. (2013) who identified ThT S-positive TDP-43 aggregates in most of the tissues from ALS and FTLD patients (Bigio et al., 2013; Robinson et al., 2013). Other researchers reported the full-length protein in inclusion bodies when expressed in *E. coli* (Fang et al., 2014) and in cytosolic aggregates when expressed in yeast (Johnson et al., 2008). In both cases, no evidence of amyloid-like structures was found and the protein did not react to ThT or Congo Red. To add complexity to the problem, Wang et al. (2013) reported that the C-terminus of TDP-43, by itself or linked to RRM2, can form well-ordered fibrillar structures that, nevertheless, do not bind amyloid-specific dyes or antibodies (Zhang et al., 2009; Wang et al., 2013). We showed that the isolated RRM1 and the two tandem RRM domains display amyloid-like features. Both RRM1 and RRM1-2 aggregates, in addition to enriching their β-sheet content, bind ThT, although RRM1 precipitation limits the quantification of the structures formed. The ability to bind the amyloid-specific dye was detected both under stressful conditions (heat treatment) and under near-to-physiological conditions (neutral buffer, 37°C). Morphological analysis by AFM suggested that, especially for RRM1-2, fibrillar features can be identified within the amorphous aggregates. These are thinner filaments joining larger, more disperse structures. This evidence strongly suggests a role of the tandem RRM domains not only in leading TDP-43 to aggregate, but also in conferring to the aggregates amyloid-like properties.

Finally, we observed quite different RNA-binding properties of the two domains: RRM1 binds to RNA with high affinity whereas we did not observe appreciable RNA-binding from RRM2. A determinant role of RRM1 in RNA recognition was also proven by Buratti and Baralle (2001) who showed that removal of RRM1 completely abolishes RNA binding, while the absence of RRM2 led to the formation of different, high-molecular weight complexes, compared to the ones produced by TDP-43 wild type. The work of these authors demonstrated that RRM1 fulfills most of the requirements necessary for RNA binding, but correct complex formation can only occur in the presence of both RRM domains. Our BLI studies supported these findings: we showed that RRM1 binds to AUG12 with a *K*_d_ in the high-nM range, while RRM2 binds the same sequence with a *K*_d_ > 30 μM. The *K*_d_ decreases to 4 nM when RRM2 is linked to RRM1 indicating a strong cooperativity between the two domains. This is reasonable since the two motifs must have evolved to optimize nuclei acid recognition, RRM1 by establishing the highest number of contacts with the nucleic acid sequence, RRM2 by stabilizing RRM1 optimal conformation and orientation. The work by Lukavsky et al. (2013) supports this hypothesis (Lukavsky et al., 2013). The authors published the NMR structure of the two tandem RRM domains of TDP-43 bound to the same single stranded RNA AUG12 used in our study and showed how RNA binds to a positive groove of the surface. The authors demonstrated how RNA-binding induced stabilizing inter-molecular interactions. Among these, they recognized the primary role of a central guanine, which interacts with both RRM1 and RRM2, and the importance of the linker in stabilization. It is thus possible that, in the absence of a cognate RNA, the 15-amino acid linker and the tendency of RRM1 toward aggregation may lead to unstable intermediates that favor formation of amyloid-like structures. This explanation would explain why we did not observe appreciable precipitation of RRM1 or RRM1-2 in our BLI studies which used RNA bound to the surface.

A direct consequence of these results is the working hypothesis that RNA could be used to increase protein solubility and prevent aggregation. If TDP-43 solubility could be influenced by its cognate nucleic acids but not by its protein binding partners, it is possible that the RRM motifs need stabilization from nucleic acids to preserve their monomeric state. This concept is supported by the study by Huang et al. (2013), who found an *in vitro* increment of TDP-43 solubility in the presence of the cognate single stranded DNA/RNA, but not when the protein hnRNP was present in the mixture. It also agrees with our previous observations that natural partners of a protein can be used to compete out aggregation which indicates aggregation as the “dark side” of non-pathological function (Pastore and Temussi, 2012). Our studies thus open a new direction for the search of a treatment of TDP-43 related diseases.

## Author Contributions

EZ performed protein construct production and characterization, including CD and ThT-binding assays, and drafted the manuscript together with AP. SM supported EZ in BLI experiments and resulting the data analysis. RT acquired the AFM micrographs. AP developed the concept behind the project together with EZ, coordinated the research, and offered support and supervision. All authors have reviewed and approved the final version of this manuscript.

## Conflict of Interest Statement

The authors declare that the research was conducted in the absence of any commercial or financial relationships that could be construed as a potential conflict of interest.
